# Distinct dimensions of interoception modulate the link between cardiovascular reactivity and perceived stress

**DOI:** 10.1186/s40101-026-00440-y

**Published:** 2026-08-01

**Authors:** Yuto Tanaka, Kazushi Shinagawa, Koki Tsuji, Satoshi Umeda

**Affiliations:** 1 Keio University Global Research Institute, Tokyo, Japan; 2 Japan Society for the Promotion of Science, Tokyo, Japan; 3 Department of Psychology, Keio University, Tokyo, Japan

**Keywords:** Interoception, Perceived stress, Cardiovascular reactivity, Hemodynamic profile, Speech task, Emotion

## Abstract

Perceived stress, even when the stressor is the same, varies across individuals. Interoception—the ability to perceive and process internal bodily states—plays a crucial role in shaping the relationship between perceived stress and physiological changes in the body. We aimed to determine how interoception modulates the translation of cardiovascular reactivity into perceived stress during various stress situations. To examine this effect under varying degrees of external pressure, participants performed a speech task under two different themes: a higher-stress condition, in which they were informed that their content about social and political issues would be evaluated by professionals, and a lower-stress condition, in which they could speak freely about their personal experiences. During the tasks, we measured cardiovascular reactivity by using the hemodynamic profile (HP), which reflects the qualitative nature of blood pressure responses (i.e., myocardial-dominant vs. vascular-dominant). Following each task, participants reported subjective emotional states. To assess individual differences in interoception, we employed a Heartbeat Counting Task (HCT) and the Multidimensional Assessment of Interoceptive Awareness (MAIA). The results revealed that, in the higher-stress condition, excitement was negatively associated with HP among individuals with higher HCT scores, whereas it was positively associated with HP among those with lower HCT scores. In contrast, in the lower-stress condition, MAIA scores interacted with HP to affect excitement, which was negatively associated with HP among individuals with higher MAIA scores, and positively associated with HP among those with lower MAIA scores. We show that the interplay between interoception and cardiovascular reactivity substantially influences the perception of excitement as a subjectively positive aspect. These results provide important insights into the quality of perceived stress, highlighting its dependence on context and individual traits. Our findings offer a physiological anthropological insight into the mind–body connection, suggesting that interpreting cardiovascular reactivity as allostasis demonstrates how individuals utilize multidimensional interoceptive cues to adapt to environmental demands and reshape their physiological states into qualitative emotional experiences.

**Supplementary Information:**

The online version contains supplementary material available at https://doi.org/10.1186/s40101-026-00440-y.

## Introduction

Individuals differ in terms of their perceptions of events, culminating in their emotional states varying across similar contexts [39]; these differences are also physiological, as seen in individuals responding differently to various stressful scenarios, e.g., speaking in front of a large audience [2, 24]. Therefore, subjective emotional states emerge from a complex interplay of psychological and physiological factors. Previous studies have established associations among various individual characteristics, physiological states, and subjective emotional states [4, 40], but the potential influence of individual characteristics that may indicate sensitivity to physiological states has not yet been fully elucidated. Given this complexity, to understand these individual differences, it is essential to examine the relationship between physiological responses and specific emotional states within a robust theoretical framework.

Stress is a representative example of such emotional states, characterized by its strong dependency on physiological processes. Definitions and classifications of stress vary widely in the field of psychology, but there is consensus on stress altering cardiovascular dynamics. Specifically, it alters various factors associated with the cardiovascular system, including heart rate, blood pressure, and hormonal and endocrine activity. Obrist [28] proposed a well-known model indicating that myocardial and vascular activities vary based on the controllability of events; myocardial responses dominate during controllable events, whereas vascular responses predominate in uncontrollable events. This framework suggests that subjective stress experiences may not simply reflect the magnitude of cardiovascular activation but may instead depend on the specific hemodynamic pattern underlying that activation. In the present study, we use the term perceived stress to refer to the subjective experience of stress-related physiological arousal during the stressor task. This experience is not treated as a direct readout of bodily signals, but rather as an interpreted subjective state arising in association with autonomic nervous system activation. This framing highlights the need to consider individual differences in how bodily states are perceived and evaluated.

Interoception encompasses the perception, processing, and integration of internal bodily states at conscious and unconscious levels [22]. Afferent signals from the cardiovascular system play a key role in modulating emotional and cognitive processes. Baroreceptors transmit cardiovascular interoceptive signals to the brain via the vagus and glossopharyngeal nerves [7]. This afferent feedback provides an important source of interoceptive information that may contribute to subjective stress. Previous studies have suggested that individual differences in interoception are associated with various cognitive processes, including emotional recognition [6, 15, 37], decision-making [10], future-oriented thinking and prospective memory [21, 38], body ownership [42], and mind wandering [34]. Furthermore, in the context of stress-related disorders, the severity of their symptoms has been linked to individual differences in interoception [32]. For example, when individuals encounter situations that induce perceived stress, such as exposure to a fearful scene, afferent signals from the body can influence perceived stress [16]. Given the expanding body of research on interoception, it is reasonable to hypothesize that individual differences in interoception interact with distinct cardiovascular response patterns to shape perceived stress.

The present study aimed to address the discrepancy between the objective measure of stress represented by cardiovascular reactivity and subjectively perceived stress by examining the interactive role of interoception. Specifically, we administered speech tasks as stressors and evaluated the underlying cardiovascular response patterns (myocardial vs. vascular) during these tasks. The subjective emotional states reported immediately following the tasks were defined as perceived stress. By analyzing these variables, we investigated how individual differences in interoception modulate the relationship between cardiovascular response patterns and perceived stress. To capture the complexity of this interaction, we focused on two critical factors: the multidimensionality of interoception (objective accuracy vs. subjective sensibility) and the context of the stressor (high vs. low stress).

First, to test this hypothesis, it is essential to distinguish the various dimensions of interoception, as the concept encompasses a broad range of constructs. According to [17], interoception is not a unitary construct but consists of distinct dimensions, such as interoceptive accuracy (IAcc; objective performance) and interoceptive sensibility (IS; subjective self-evaluation). To examine how these specific aspects differentially influence stress perception, this study assessed both dimensions using the Heartbeat Counting Task (HCT; [31]) and the Multidimensional Assessment of Interoceptive Awareness (MAIA; [27]), respectively.

Second, to address the modulatory effect of interoception on the relationship between cardiovascular responses and perceived stress, we utilized a public speech task—one of the methods typically employed in stress research—to induce different types of stress. The public speech task is a standard method used in social psychology to induce psychological and physiological stress responses [23]. This task is designed to create a social evaluation threat by generating a strong stress response from the potential for negative judgment by others. Therefore, we utilized this task as a condition that induces high-level mental stress. Furthermore, when investigating how interoception affects perceived stress, it is crucial to examine what differences emerge across different situations. Thus, as a comparison to the public speech task, we designed a situation where the motor level, which is delivering a speech remained identical, but where a different level of stress load was applied. In this task, we informed participants that they would not be graded or evaluated at all and asked them to freely speak about a private topic they felt comfortable with. For convenience, we referred to this task as the private speech task. Using this comprehensive design, we aimed to examine the processes by which interoception relates to the link between cardiovascular reactivity and perceived stress.

Finally, to elucidate the relationships between interoception and perceived stress, focusing solely on the magnitude of blood pressure changes is insufficient; it is essential to evaluate the underlying hemodynamic balance. Mean arterial pressure (MAP) is maintained through a homeostatic trade-off between cardiac output (CO) and total peripheral resistance (TPR; [28]). However, traditional analytical approaches often force individuals into discrete categories, thereby precluding a nuanced evaluation of the continuous compensatory balance between myocardial and vascular activities. To overcome this limitation, we adopted the hemodynamic profile (HP; [18, 19]). This framework provides two continuous indices: HP, which reflects the qualitative balance of whether blood pressure changes are driven primarily by myocardial effort or vascular resistance, and compensatory deficit (CD), which quantifies the efficiency of the homeostatic regulation. HP represents an objective physiological index derived from cardiovascular activity, whereas interoception was conceptualized as a moderating factor that may influence how cardiovascular reactivity is associated with subjective stress experience. By using these continuous measures, we aimed to capture subtle shifts in cardiovascular reactivity and examine how they relate to perceived stress through individual differences in interoception.

To examine how task-evoked physiological responses and interoceptive processes jointly shape perceived stress, we measured continuous blood pressure in addition to heart rate during speech tasks of varying stress loads. In the stressor tasks, physical demands were held constant while social-evaluative elements were manipulated to produce variations in stress load. We adopted the hemodynamic profile/compensation deficit (HP/CD) model, which enables the myocardial-vascular balance of blood pressure regulation to be characterized on a continuous spectrum, rather than a categorical variable. Individual differences in interoception were assessed along two distinct dimensions: objective accuracy and subjective sensibility. This design allows us to explore the pathways through which stressor-elicited physiological response patterns, in concert with interoceptive individual differences, are associated with the experience of perceived stress. We formulated two distinct hypotheses regarding how individual differences in interoception modulate the relationship between cardiovascular profiles and subjective stress evaluations. First, we hypothesized that IAcc, measured via HCT, would moderate the qualitative alignment between physiological signals and subjective appraisal. For individuals with higher IAcc, subjective evaluation was expected to align more closely with the qualitative nature of the cardiovascular state; thus, a vascular-dominant response was expected to inhibit positive appraisal. Second, we hypothesized that IS, measured via MAIA, would moderate the intensity rather than the direction of subjective stress experience. That is, individuals with higher IS were expected to experience stronger subjective stress reactions triggered by physiological arousal, regardless of whether the underlying response was vascular- or myocardial-dominant. Taken together, we predicted that IAcc and IS would uniquely moderate the translation of cardiovascular reactivity into perceived stress through distinct pathways.

## Methods

### Participants

Since we plan to conduct a linear multiple regression analysis with three predictors, we performed an a priori power analysis for sample size estimation. We performed an a priori power analysis for a linear multiple regression using G*Power [12]. The assumed effect size was set to Cohen’s *f*^2^ = 0.30. This value was chosen as a relatively large regression effect based on related studies reporting medium-to-large associations between interoceptive measures and subjective outcomes, as well as moderation effects involving interoceptive measures and physiological responses [13, 26, 41, 30]. With an alpha level of 0.05 and power of 0.80, the required minimum sample size was 41 participants. A total of 44 undergraduate students (10 males and 32 females) from Keio University participated in the experiment, with a mean age of 21.42 years (SD = 2.78). Prior to the experiment, participants were screened to ensure they did not have any cardiovascular or neurological conditions and psychiatric disorders, including social anxiety. This study was approved by the Keio University Research Ethics Committee (No. 23109), and all experiments were conducted in accordance with the relevant guidelines and regulations. Three participants were excluded from all subsequent analyses due to data loss during the recording process, resulting in a final sample size of 41 participants included in the analysis.

### Apparatus

The NIBP100E (Biopac) was utilized to measure continuous blood pressure. The device was equipped with cuffs on the left index finger, middle finger, and upper arm. Data recording and raw data extraction were conducted using AcqKnowledge 5.0 (Biopac). Further statistical analyses were carried out using the open-source statistical software R (version 4.3). A pulse oximeter (NONIN, Model 3012LP XPOD, Model 8000SL) was employed to perform the Heartbeat Counting Task.

### Procedures

The participants signed the informed consent form and completed the State-Trait Anxiety Inventory (STAI). The cuff for the noninvasive continuous blood pressure monitoring system was then placed on the left index and middle fingers, as well as on the upper arm. Participants engaged in public and private speech tasks, with the order of tasks counterbalanced among participants. Upon completion of both tasks, the cuff was removed, a pulse oximeter was applied, and the Heartbeat Counting Task was administered. The entire experiment lasted 90 min and included an explanation for the participants.

### Questionnaires

To assess task-evoked subjective emotional states related to perceived stress during the speech task, we developed a 10-item questionnaire asking participants how they felt during the task. In line with our operational definition of perceived stress as the subjective experience of stress-related physiological arousal, the items were selected to capture subjective emotional states associated with physiological activation during the speech task. Specifically, items reflecting positively valenced activation or engagement, such as enjoyment and active involvement, were labeled excitement, whereas items reflecting negatively valenced tension or threat-related arousal, such as anxiety and tension, were labeled nervousness. These labels were not intended to equate all positive emotions with excitement or all negative emotions with nervousness. Rather, they were used as concise descriptors of two task-evoked subjective emotional components associated with stress-related physiological arousal in the present speech-task context. Thus, excitement and nervousness were treated as complementary indices of task-evoked subjective arousal associated with perceived stress, rather than as comprehensive measures of positive and negative emotion. We aggregated the scores from each item to quantify these dimensions. The questions are provided in Japanese and English in the Supplementary Materials. To evaluate the internal consistency of the newly used measures for excitement and nervousness, we calculated Cronbach’s *α* coefficients. The results showed that the internal consistency was *α* = 0.86 for excitement and *α* = 0.87 for nervousness. We also assessed the Japanese version of the Multidimensional Assessment of Interoceptive Awareness (MAIA; [35]) as a measure of IS. For the questionnaires, the total scores for each were used.

State anxiety is closely related to stress responses during speech tasks and may influence the link between bodily awareness and subjective emotional evaluation. Previous research has shown that anxiety is associated with perceived stress [9] and individual differences in interoception [29]. We therefore measured state anxiety using the State-Trait Anxiety Inventory-State form (STAI-S; [33, 36]) and included it as a control variable in the regression models.

### Speech task

In this study, we employed a public speech task, a conventional method utilized as a stressor. This experimental procedure reliably induces changes in physiological responses by increasing the stress load [1, 11]. We attempted to induce different types of stress while controlling physical conditions and motor demands. The order of the speech tasks was counterbalanced among participants.

Participants were required to deliver a speech on a pre-assigned, challenging social issue which could not be freely chosen. They were informed in advance that their performance would be video-recorded and subsequently evaluated. A live video feed was displayed on a screen positioned directly in front of them, allowing them to observe themselves in real-time.

Five current social and political topics were prepared for public speeches. We utilized questions from a Japanese-English proficiency test, which were originally written in English and subsequently translated into Japanese by the authors. They were given one minute to prepare before their speeches and were advised that their presentations would be videotaped and later evaluated by experts. The recorded data were not assessed and were discarded at the conclusion of the experiment. Following the instructions, participants ranked five topics in order of ease for a three-minute speech without being informed of the specific topic they would discuss.

Three minutes after measuring continuous blood pressure as part of the pre-speech phase, participants were notified that they would deliver a speech on the topic they had identified as the third easiest to articulate. The third easiest topic was selected for the high-stress condition to ensure a sufficient stress response through increased task challenge while maintaining task compliance; using the most difficult topic could have hindered participants’ ability to speak continuously for the full three minutes. In contrast, the easiest topic was assigned to the low-stress condition to serve as a comparative baseline with minimal psychological load. They were allowed one minute to contemplate the topic before delivering a three-minute speech, which was displayed on a computer monitor. Due to the potential fluctuations in blood pressure related to their speaking activities, participants were instructed to speak for a full three minutes. Upon completion of their speeches, participants filled out a questionnaire regarding their emotional states during the task.

To simulate a less stressful condition, participants engaged in one minute of preparation followed by three minutes of speech; however, the speeches were not recorded during the task. Participants ranked six speech topics in order of ease for a three-minute speech without being informed of the specific topic they would discuss. Three minutes after measuring continuous blood pressure, participants were informed that they would deliver a speech on the topic they had identified as the easiest to discuss. After a one-minute preparation period, participants delivered their speeches for three minutes, during which continuous blood pressure measurements were again recorded. Following the completion of the speeches, participants filled out a questionnaire regarding their feelings during the task.

### Heartbeat Counting Task (HCT)

Participants placed a pulse oximeter on their left index finger. In the HCT, we instructed participants to silently count their heartbeats at different intervals without checking their pulses or altering their breathing. Participants were reminded to report the number of times they actually felt a heartbeat rather than making an estimation. This task was repeated six times with time intervals of 25, 30, 35, 40, 45, and 50 s, presented in a randomized order. Participants began counting at the sound of “start” and concluded counting at the sound of “stop” for each interval. After counting, they reported the number of heartbeats they counted, and their confidence in their responses was assessed using a keyboard. This task was conducted using the “cardioception” Python package [25]. The absolute proportional difference between the number of reported and actual heartbeats was calculated to quantify the HCT scores (Eq. 1). The average of the 6 trials was treated as the heartbeat counting score.

1 $$100* \frac{1}{6}\sum{1}-\frac{\left|Actual-Reported\right|}{Actual}$$

### Data acquisition

Continuous physiological measures—systolic blood pressure (SBP), diastolic blood pressure (DBP), mean blood pressure (MBP), heart rate (HR), and stroke volume (SV)—were recorded and processed using AcqKnowledge software. For the analysis, the data were downsampled to 250 Hz and converted to pulse cycle units. Cardiac output (CO) and total SV were estimated using AcqKnowledge, with cardiac output derived from arterial blood pressure (ABP). We used the default initial cardiac output values provided by the AcqKnowledge software (BIOPAC Systems, Inc.): 5.6 L/min for males and 4.9 L/min for females. Total peripheral resistance (TPR) was calculated manually using MBP and CO.

To exclude intervals with unsettled physiological states from the analysis, specific periods were included for each resting and task phase. The data, converted to pulse cycle units, were divided into ten sections based on the total number of cycles. Although the total number of cycles varied with participants’ heart rates, the elapsed time per section remained approximately consistent across participants. Since the pre-speech phase served as baseline data, we excluded two sections: one immediately following the measurement and the other just before the transition to the next session, during which the data tended to be unstable. Task phase averages were calculated by excluding the first two sections, as many participants exhibited disturbed physiological states shortly after the start.

To remove extreme outliers, we excluded cycles that met any of the following criteria for extreme outliers: physiologically implausible values, defined as HR outside 45–200 bpm or blood pressure (SBP, DBP, and MBP) outside 50–250 mmHg; or logical errors resulting from mechanical artifacts, defined as CO or TPR values of zero or less. Following the removal of these outliers, we computed the averages and standard deviations for each condition and participant, excluding any cycles that exceeded three standard deviations as outliers.

To quantify cardiovascular reactivity during the task in comparison to baseline levels, we calculated the logarithm of the rate of change from baseline to task performance. In accordance with Gregg et al. [19], we rotated the two-dimensional space formed by the logarithmic change rates of CO and TPR by 45 degrees to derive the HP and CD values.

### Statistical analysis

We performed multiple regression analyses on the two subjective emotional evaluation factors obtained from each of the two tasks. Each model included the HP and the two interoceptive indices (HCT score and MAIA) as main effects, along with all possible two-way and three-way interactions among these three variables. The STAI-S state anxiety score was included as a control variable. To control for potential task order effects, the order of tasks was counterbalanced across participants. Therefore, task order was not included as an independent variable in the statistical model. The primary analyses in the main text thus comprised four models, with additional analyses involving the CD index reported in the Supplementary Materials.

We performed centering to mitigate multicollinearity by subtracting the mean value from each independent variable. This process is widely recommended to reduce multicollinearity among independent variables and their constructed interactions. Additionally, we employed the variance inflation factor (VIF) as an indicator of multicollinearity in our multiple regression analyses. This was calculated by regressing each independent variable against all other independent variables in the model.

We estimated the parameters of the statistical models using a Bayesian approach with the BRMS package in R [3]. An uninformed uniform distribution was employed as the prior distribution to mitigate bias from prior information regarding the effects of independent variables. Student’s *t*-distribution was utilized for the intercept and variance parameters, which were set automatically based on the default settings of the BRMS package. The Markov Chain Monte Carlo (MCMC) method was employed for parameter estimation, with each chain comprising 2000 samples and 1000 burn-in samples, resulting in a total of 1000 posterior samples per chain, or 4000 samples for each model parameter. We verified the validity and reliability of the parameter estimation results by assessing the effective sample size and R-hat for each parameter.

## Results

### Self-reported results

First, we compared the perceived stress of the speeches on the participants. Table 1 presents the statistics of the subjective emotional evaluations and the results of the comparison. When comparing subjective emotional evaluations across tasks, a trend was observed indicating higher ratings for excitement in private speech and higher ratings for nervousness in public speech. Table 2 outlines the personal characteristics of the participants.

**Table 1:** Comparison of subjective emotional evaluation across public and private speaking

Emotion	Public speaking: mean (SD)	Private speaking: mean (SD)	*t*-value	df	*p*-value
Excitement	11.78 (4.27)	14.54 (4.11)	− 2.98	79.88	0.004
Nervousness	15.02 (4.74)	12.68 (4.80)	2.22	79.99	0.029

*SD *standard deviation, *df *degrees of freedom

**Table 2:** Participant characteristics across tests

Variables	Mean	SD
HCT	53.68	25.12
MAIA	91.63	21.56
STAI-S	41.27	5.60

*HCT *Heartbeat Counting Task score, *MAIA *Multidimensional Assessment of Interoceptive Awareness, *STAI-S *State-Trait Anxiety Inventory (State)

### Physiological measures

Table 3 illustrates the mean values of the various physiological measures for each speech task. There were significant differences between private speech and public speech in HR (*t*(40) = − 2.32, *p* = 0.03), SBP (*t*(40) = − 2.12, *p* = 0.04), DBP (*t*(40) = − 2.16, *p* = 0.04). No other significant differences were observed between conditions. Although MBP did not reach the conventional significance level (*p* = 0.05), the direction of the effect was consistent with SBP and DBP. Overall, these basic physiological measures indicate that the public speech condition was associated with a relatively higher level of cardiovascular reactivity compared to the private speech condition.

**Table 3:** Difference in physiological measures across public and private speaking

	Speech condition				
	Private		Public		*p*-value
HR (bpm)	88.39	(12.23)	93.10	(18.20)	0.03
SV (mL)	74.02	(14.88)	70.98	(18.25)	0.41
SBP (mmHg)	123.71	(20.95)	127.38	(20.53)	0.04
DBP (mmHg)	80.44	(16.81)	83.07	(15.38)	0.04
MBP (mmHg)	96.93	(17.12)	99.40	(15.92)	0.05
CO (L/min)	6.82	(1.00)	7.03	(1.28)	0.47
TPR (mmHg·min/L)	14.68	(3.93)	14.61	(3.57)	0.77
HP	0.01	(0.20)	0.02	(0.21)	0.74
CD	0.11	(0.08)	0.13	(0.08)	0.24

*HR *heart rate, *SV *stroke volume, *SBP *systolic blood pressure, *DBP *diastolic blood pressure, *MBP *mean blood pressure, *CO *cardiac output, *TPR *total peripheral resistance, *HP *hemodynamic profile, *CD *compensation deficit

### Public speech (higher stress condition)

We utilized the variance inflation factor (VIF) as a measure of multicollinearity in our multiple regression analyses. The maximum VIF value observed was 1.209, which is significantly below the stringent threshold of 4, indicating that multicollinearity is unlikely to have influenced the results. We examined the effects of cardiovascular reactivity and individual differences in interoception on subjective emotional evaluation during a speech task, regressing out the potential influence of task-induced state anxiety.

The effects on the evaluation of excitement and nervousness during the public speech task are presented in Table 4. Notably, the *R*-hat values for each parameter estimation approached 1, and the effective sample size (ESS) was adequate, thereby supporting the reliability and validity of the current estimations.

**Table 4:** The interactive effects of hemodynamic profile and interoception on the evaluation of excitement and nervousness during the public speech task

	Excitement				Nervousness			
Parameters	Estimate	95% CI	*R*	ESS	Estimate	95% CI	*R-hat*	ESS
Intercept	11.70	[10.50, 12.90]	1	5036	15.49	[14.02, 16.92]	1	3892
HP	1.05	[− 5.24, 7.44]	1	3703	2.25	[− 6.27, 10.78]	1	3494
HCT	− 0.00	[− 0.06, 0.05]	1	3692	0.02	[− 0.05, 0.08]	1	4160
MAIA	0.05	[− 0.01, 0.12]	1	4177	− 0.02	[− 0.10, 0.05]	1	4168
STAI-S	0.39	[0.18, 0.60]	1	4521	− 0.30	[− 0.55, − 0.04]	1	4594
HP:HCT	− 0.36	[− 0.72, − 0.01]	1	4288	− 0.16	[− 0.62, 0.30]	1	3553
HP:MAIA	0.04	[− 0.37, 0.45]	1	3156	0.08	[− 0.43, 0.59]	1	3742
HCT:MAIA	0.00	[− 0.00, 0.00]	1	4054	− 0.00	[− 0.00, 0.00]	1	4057
HP:HCT:MAIA	0.01	[− 0.02, 0.03]	1	3707	0.01	[− 0.01, 0.04]	1	3572

*HCT *Heartbeat Counting Task score, *MAIA *Multidimensional Assessment of Interoceptive Awareness, *STAI-S *State-Trait Anxiety Inventory (State), *HP *hemodynamic profile, *CD *compensation deficit, *95% CI *95% credible intervals, *ESS *effective sample size

**Fig. 1:**
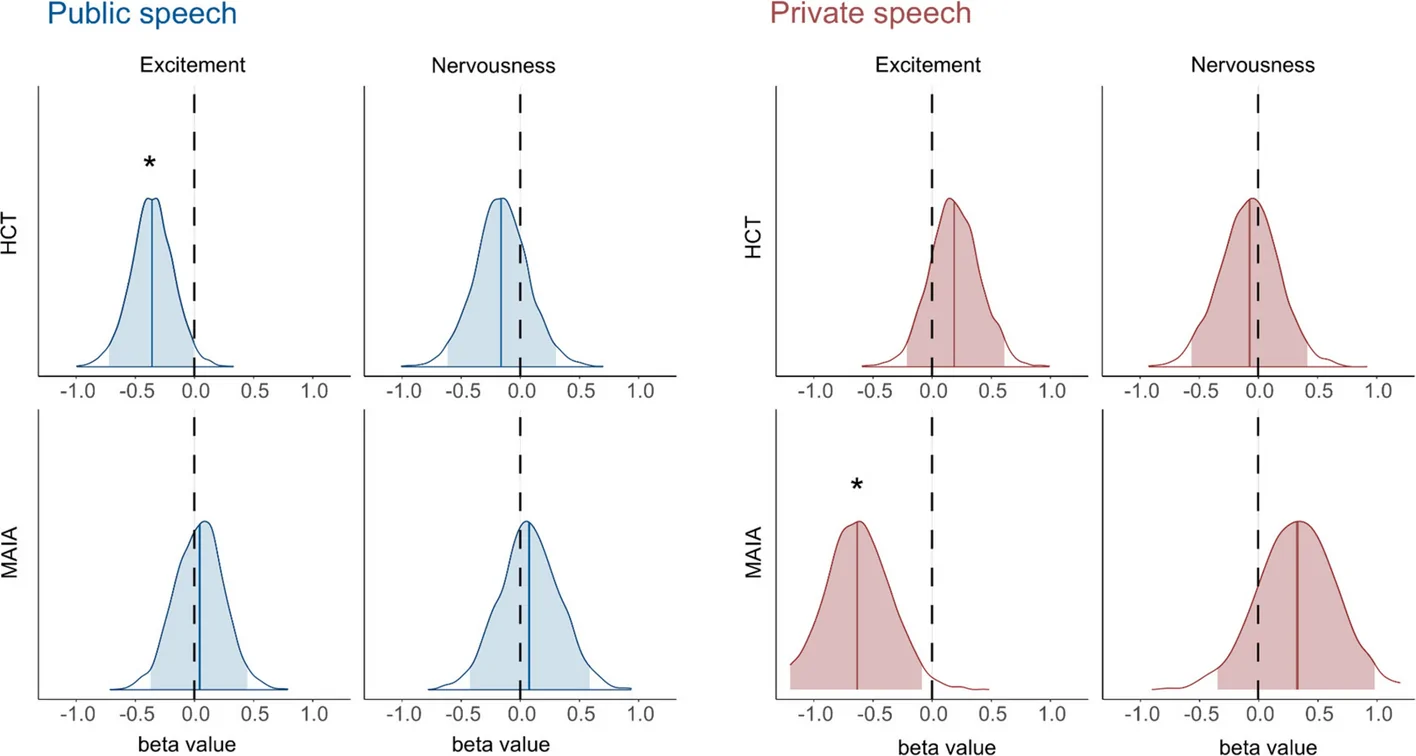
Distributions of estimated beta parameters of interactions between cardiovascular reactivity and interoception on excitement or nervousness during each task condition. Dark lines indicate the mean of the estimated beta value, and the light blue ranges indicate 95% credible intervals (CI). Asterisks indicate that the 95% CI did not include 0

**Fig. 2:**
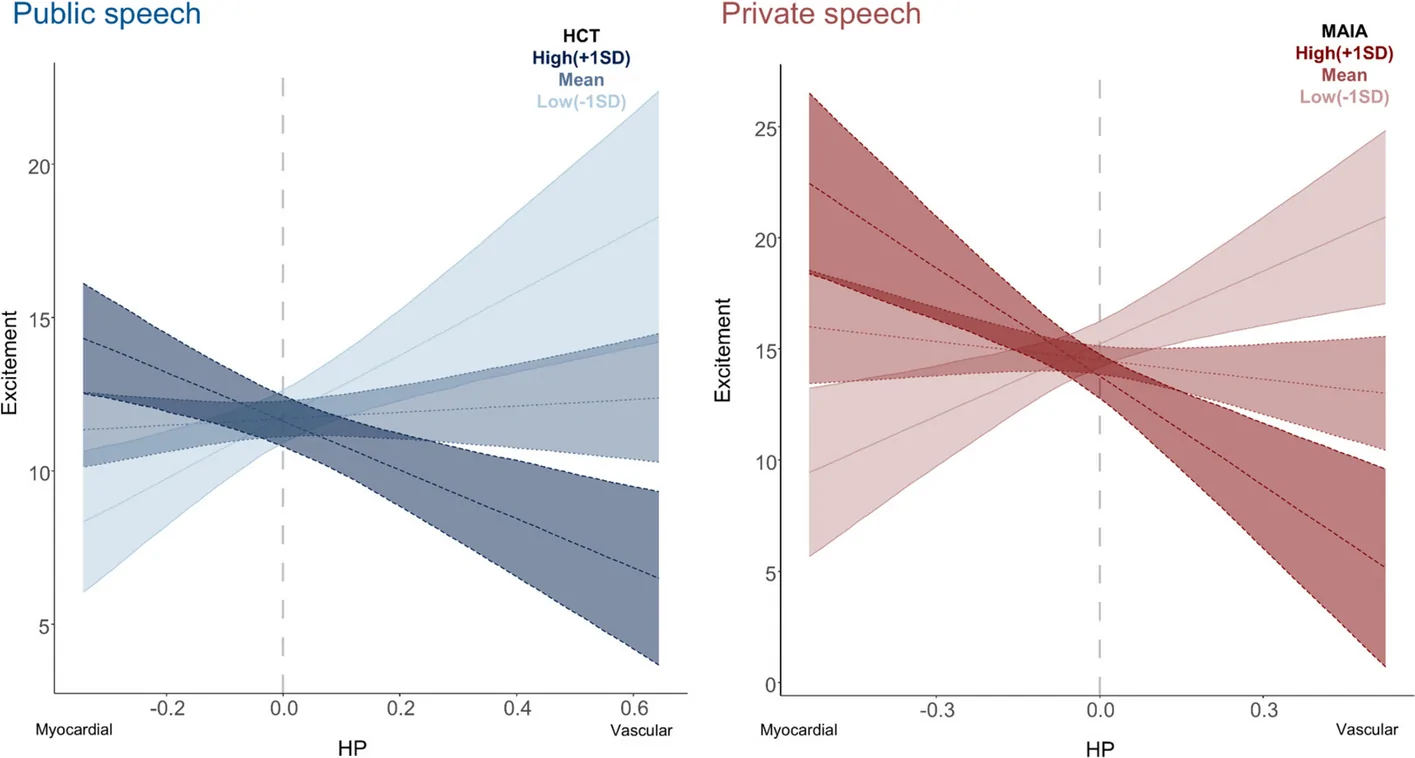
Effect of interactions between cardiovascular reactivity and interoception on excitement or nervousness during each speech task. The vertical axis represents the score for each emotional evaluation, and the horizontal axis indicates the value for hemodynamic profiles (HP). Colors indicate the relative levels of each interoceptive measure (Heartbeat Counting Task score: HCT; Multidimensional Assessment of Interoceptive Awareness: MAIA). The range indicates the standard error

Among the effects on excitement evaluation during public speech, we observed a credible interaction between cardiovascular reactivity (HP) and HCT score, as well as a credible main effect of state anxiety (Table 4). For these parameter estimates, the 95% credible interval (95% CI) of the posterior distribution did not include zero, indicating a meaningful effect on the dependent variable. We illustrated the estimated interaction effects in Fig. 1. Given the observed interaction effect, we further examined the relationship by plotting the HCT scores based on their relative values (Fig. 2). The relatively high state is designated as mean + 1 SD, whereas the low state is defined as mean − 1 SD. The results indicate that higher HCT scores, referred to as higher IAcc, were associated with lower excitement ratings at higher HP values. Additionally, although the positive effect of STAI-S influenced the evaluation of excitement, a negative effect of this scale was also observed for nervousness.

### Private speech (lower stress condition)

The effects on the evaluation of excitement and nervousness during the private speech task are presented in Table 5. Notably, the *R*-hat values in each parameter estimation approach 1, similar to the findings from the public speech task, and the effective sample size (ESS) is adequate, which support the reliability and validity of the current estimations.

**Table 5:** The interactive effects of hemodynamic profile and interoception on the evaluation of excitement and nervousness during the private speech task

	Excitement				Nervousness			
Parameters	Estimate	95% CI	*R*	ESS	Estimate	95% CI	*R-hat*	ESS
Intercept	14.49	[13.21, 15.77]	1	4385	12.60	[10.96, 14.21]	1	4757
HP	− 2.83	[− 12.24, 6.49]	1	2650	− 3.31	[− 14.51, 7.88]	1	3476
HCT	0.01	[− 0.05, 0.07]	1	3457	0.05	[− 0.02, 0.11]	1	4324
MAIA	− 0.03	[− 0.11, 0.04]	1	3220	0.02	[− 0.06, 0.11]	1	3723
STAI− S	0.22	[− 0.02, 0.45]	1	3091	− 0.04	[− 0.32, 0.25]	1	4450
HP:HCT	0.19	[− 0.21, 0.61]	1	2528	− 0.07	[− 0.57, 0.42]	1	3286
HP:MAIA	− 0.63	[− 1.20, − 0.08]	1	2656	0.33	[− 0.34, 0.98]	1	3814
HCT:MAIA	0.00	[− 0.00, 0.00]	1	4180	0.00	[− 0.00, 0.01]	1	4742
HP:HCT:MAIA	0.01	[− 0.01, 0.02]	1	2650	0.02	[− 0.00, 0.04]	1	3596

*HCT *Heartbeat Counting Task score, *MAIA *Multidimensional Assessment of Interoceptive Awareness, *STAI-S *State-Trait Anxiety Inventory (State), *HP *hemodynamic profile, *CD *compensation deficit, *95% CI *95% credible intervals, *ESS *effective sample size

In terms of the effects on excitement evaluation during private speech, we identified a credible interaction between cardiovascular reactivity (HP) and MAIA score (Table 5). For these parameter estimates, the 95% credible interval (95% CI) of the posterior distribution did not include zero, indicating a substantial effect on the dependent variable. Given the presence of an interaction effect, we plotted MAIA based on its relative values to explore the relationship in greater detail (see Fig. 2). The relatively high state is defined as mean + 1 standard deviation (SD), and the low state as mean − 1 SD. The results indicated that when MAIA was high, higher HP values were correlated with lower excitement ratings compared with lower HP values. The credible intervals for all independent variables included zero regarding the evaluation of nervousness.

## Discussion

This study aimed to investigate the influence of cardiovascular reactivity on perceived stress in situations of high physiological arousal, as well as the relationship with interoception. The results indicated that, even after controlling individual characteristics affecting the speech task, differences in interoception modulated the impact of cardiovascular reactivity on subjective emotional states during the task. Specifically, individuals with higher IAcc reported greater excitement at lower HP and lower excitement at higher HP, whereas the opposite pattern was observed among individuals with lower IAcc. The findings of this study suggest that perceived stress is influenced by physiological states and interoception. Previous research has noted that interoception affects emotional recognition [15] and perceived stress [32]. The results of this study are consistent with these prior findings. Furthermore, this study demonstrated that the direction of the differences in HP varied according to interoception, indicating that cardiovascular reactivity does not directly correspond to subjective emotional states; rather, individual differences in interoception modulate the experience of perceived stress.

A comparison of subjective emotional evaluations revealed greater nervousness and lower excitement during the public speech task than during the private speech task (Table 1). Differences were also observed between tasks in HR and basic blood pressure indices; specifically, SBP and DBP were higher during public speech (Table 3). Although the two task conditions were matched in terms of physical demands, with both requiring participants to speak for a fixed duration, their subjective emotional states and cardiovascular reactivity differed. These findings indicate that the task designs elicited affective and physiological states corresponding to qualitatively different stressors.

In the public speech task, a credible interaction was observed between HP and HCT scores regarding the subjective evaluation of excitement (Table 4). For individuals with higher HCT scores, HP was negatively associated with excitement, whereas a positive association was observed for those with lower HCT scores. This suggests that the relationship between cardiovascular reactivity and subjective stress evaluation is not uniform, but is moderated by individual differences in IAcc. An increased HP value indicates that the contribution of TPR to blood pressure change is relatively large, reflecting a more vascular-dominant response. Previous research has associated such vascular responses with situational uncontrollability or threat states. As a tentative interpretation regarding the underlying psychological factors, this physiological signal of uncontrollability may be linked with the subjective evaluation, likely impeding the subjective evaluation of excitement in individuals with higher interoceptive accuracy (High HCT). Conversely, among individuals with lower HCT scores, the subjective evaluation showed the opposite direction. Although the underlying psychological mechanism remains unclear, our results demonstrate a statistical association showing that individual differences in interoceptive traits modulate how physiological stress responses relate to subjective experiences. However, the reliability of the HCT as a measure of individual differences in interoception remains under debate [43].

In the private speech task, participants with higher IS reported lower subjective excitement when their cardiac state was more vascular. This suggests that individual differences in overall IS modulate the subjective experience of self-disclosure. Unlike the public speech task, which involves external evaluation, private speech is inherently self-referential. As a tentative interpretation regarding the underlying evaluative processes, when these individuals experienced high HP, which signifies a physiological “threat” or “avoidance” state, they may link this internal signal to their evaluation of the task. Consequently, they reported lower excitement, perceiving the high-vascular state as inconsistent with a positive emotional experience. Conversely, individuals with lower IS, who may be less attuned to the qualitative nuances of their cardiovascular state, might have relied more on the situational context of the task. The overall physiological arousal during self-disclosure was likely interpreted as "excitement," regardless of the underlying vascular dominance. Regarding the findings in the private speech task, the credible interaction was observed with MAIA scores rather than HCT scores. We interpret this as indicating that the private speech task, which involves self-referential processing and personal disclosure, may be more closely aligned with the self-reflective nature of MAIA, which is described as IS.

In this study, the effects of interoception and the cardiovascular system during the speech task were observed only for excitement ratings, not nervousness. This discrepancy may stem from the different functional roles of these emotional states during a speech stressor. Nervousness can be considered a primary response to speaking under pressure; thus, it may have been less sensitive to the subtle moderating effects of interoception and HP, as all participants might feel nervous in this situation. In contrast, excitement reflects a positive appraisal of high arousal that is aligned with the challenging situation. Because an increased HP value represents a physiological threat state, this specific signal may have inhibited the positive appraisal of the stressor in individuals who were highly attuned to their internal state.

Beyond the cardiovascular and interoceptive indices, state anxiety also showed a credible association with subjective evaluations during the public speech task. Specifically, higher state anxiety was associated with higher excitement and lower nervousness. These effects were not observed in the private speech task, suggesting that factors specific to the public speech context, such as tension regarding the topic and the evaluation process, may play a critical role. For individuals with higher levels of state anxiety, the high situational demands and resulting physiological arousal during public speaking might be labeled as "excitement" rather than standard "nervousness" upon task completion. The precise temporal dynamics of these anxiety-related shifts during the task require further direct assessment.

Our findings suggest that the HP-CD model could potentially serve as a helpful framework for analyzing and understanding the modulatory effect of interoception on the relationship between cardiovascular responses and perceived stress. These findings, however, should be interpreted in light of several limitations. Interoception was assessed as an individual difference using the HCT and MAIA, rather than by directly measuring real-time perception of physiological changes during the speech task. Although HCT and MAIA are commonly used to characterize individual differences in interoception, we cannot rule out a discrepancy between these trait-like measures and online interoceptive processing under high-stress conditions. Future studies should incorporate real-time measures to further elucidate these state-level dynamics.

In addition, this study primarily focused on perceived stress related to speech and did not address physical stress, such as that induced by the cold pressure test. The term "stress" encompasses a wide range of experiences, and mental and physical stress may exhibit commonalities and differences. To investigate the relationship between perceived stress, physiological states, and interoception in future research, it is essential to explore various types of stress in a novel way. Furthermore, a prior study indicated that cardiovascular reactivity and CD risk differ between African Americans and European Americans [8]. For discussions on the HP-CD model and stress reactivity in relation to the development of cardiovascular disease, refer to Howard [20] and other relevant literature. Additionally, another study found that interoceptive awareness while viewing a fear-inducing film varied between West Africans and European Americans [5], and the factors associated with the MAIA may differ between Japanese and European Americans [14]. Therefore, including our study, it is challenging to draw a definitive conclusion regarding a global integrative understanding of perceived stress, interoception, and cardiovascular reactivity.

The present research may also be considered in relation to theories of constructed emotion. These theories propose that the brain does not passively detect emotions, but instead assigns meaning to bodily states while regulating the body through allostatic control. From this perspective, psychological stress may emerge through the predictive interpretation of bodily and situational information. Future studies should therefore measure brain activity in addition to autonomic nervous activity, thereby enabling a more direct examination of the processes through which psychological stress is generated.

From the perspective of physiological anthropology, our findings contribute to a holistic understanding of the dynamic interplay between physiological regulation and psychological experience. Our study suggests that individual differences in interoception, encompassing both objective and subjective dimensions, are involved in modulating how complex autonomic states relate to subjective emotions such as excitement. Based on these empirical results, we discuss how our findings could be interpreted within a broader scope to suggest potential directions for future theoretical frameworks. It is anticipated that associations among interoception, emotions, and autonomic states can be interpreted within a broader framework of physiological anthropology and allostasis. By examining the fine-grained dynamics of autonomic function using HP-CD, future approaches may facilitate an interpretation of cardiovascular reactivity not merely as a temporary stress response, but as a component of allostasis, the process of achieving stability through physiological change. From this broader theoretical perspective, multidimensional interoception might hypothetically serve as a pivotal interface in this allostatic process, translating autonomic states into subjective experiences. However, this is merely one possible interpretation, and we are not yet at a stage to definitively narrow down the range of alternative explanations. Understanding the link between detailed physiological assessment and multidimensional interoceptive profiling is essential for deepening our understanding of the mind–body connection and for revealing how humans adaptively reshape their physiological states to cope with environmental demands.

## Conclusion

In the present study, we demonstrated that, in situations eliciting cardiovascular reactivity, the interplay between cardiovascular reactivity and individual differences in interoception was credibly associated with perceived stress. Although public speech is commonly utilized as a social and interpersonal stressor, only a limited number of studies have examined stress in this context at subjective and physiological levels, including the effects of interoception that connect these levels. Our findings indicate that, in addition to anxiety, individual differences in interoception should be considered when examining perceived stress. Moreover, our findings reveal that these relationships are influenced differently based on the topic of the speech task and the contextual setting, such as the presence of evaluative feedback. These results imply that the association between cardiovascular reactivity and perceived stress varies according to interoceptive traits (i.e., IAcc and IS) and environmental context (public vs. private speech), even when the physical act of speaking is broadly comparable across tasks. Future research should explore the influence of interoception in stressful situations beyond speech tasks, as well as contexts that evoke positive emotional states, such as sports and games, to further elucidate these influencing relationships.

## Supplementary Information

Supplementary Material 1.

## Data Availability

Data will be made available on request.
