# Supplementary material for: Distinct dimensions of interoception modulate the link between cardiovascular reactivity and perceived stress
Source: J Physiol Anthropol. 2026 Aug 1;45:26. doi: 10.1186/s40101-026-00440-y (PMC13718030; doi:10.1186/s40101-026-00440-y)
Supplement: Supplementary file 1 [file 40101_2026_440_MOESM1_ESM.docx]

# Appendix

# Speech theme of the public speaking task

Rank these topics in the order in which you feel comfortable to make the speech.

1. Do you think individual efforts change society for the better?
2. Should schooling emphasize cooperation or competition?
3. Do you think that people have the right to own firearms for defense?
4. Does science contribute more to society than art?
5. Do you think social networking has more disadvantages than profit?

# Speech theme of the private speaking task

Rank these topics in the order in which you feel comfortable to make the speech.

1. Talk about the things or events that you feel nostalgic about.
2. Talk about the events you recently enjoyed.
3. Talk about upcoming events that you are looking forward to.
4. Talk about the places you would like to visit or things you would like to do.
5. Talk about the events that you have recently done and are satisfied with.
6. Talk about your favorite food or foods that you enjoyed recently.

# Questionnaire about the speech

How did you feel while you were performing the speech? Rate from 1 (not at all) to 5 (feel at most)

| 1 | Excited | 1 | 2 | 3 | 4 | 5 |
| --- | --- | --- | --- | --- | --- | --- |
| 2 | Active | 1 | 2 | 3 | 4 | 5 |
| 3 | Enjoyed | 1 | 2 | 3 | 4 | 5 |
| 4 | Pleasant | 1 | 2 | 3 | 4 | 5 |
| 5 | Worried | 1 | 2 | 3 | 4 | 5 |
| 6 | Intense | 1 | 2 | 3 | 4 | 5 |
| 7 | Tired | 1 | 2 | 3 | 4 | 5 |
| 8 | Awaken | 1 | 2 | 3 | 4 | 5 |
| 9 | Nervous | 1 | 2 | 3 | 4 | 5 |
| 10 | Relaxed | 1 | 2 | 3 | 4 | 5 |

In Japanese

| 1 | わくわくした | 1 | 2 | 3 | 4 | 5 |
| --- | --- | --- | --- | --- | --- | --- |
| 2 | 積極的な気分であった | 1 | 2 | 3 | 4 | 5 |
| 3 | 楽しかった | 1 | 2 | 3 | 4 | 5 |
| 4 | 愉快であった | 1 | 2 | 3 | 4 | 5 |
| 5 | 心配であった | 1 | 2 | 3 | 4 | 5 |
| 6 | 気が張り詰めた | 1 | 2 | 3 | 4 | 5 |
| 7 | ぐったりした | 1 | 2 | 3 | 4 | 5 |
| 8 | 神経がたかぶった | 1 | 2 | 3 | 4 | 5 |
| 9 | 緊張した | 1 | 2 | 3 | 4 | 5 |
| 10 | ゆったりした | 1 | 2 | 3 | 4 | 5 |

**Supplementary Table 1**. The interactive effects of compensation deficit and interoception on the evaluation of excitement and nervousness during the public speech task.

|  | Excitement | | | | Nervousness | | | |
| --- | --- | --- | --- | --- | --- | --- | --- | --- |
| Parameters | Estimate | 95%CI | R | ESS | Estimate | 95%CI | R | ESS |
| Intercept | 11.46 | [10.18, 12.73] | 1 | 4032 | 15.65 | [14.15, 17.14] | 1 | 3899 |
| CD | 0.52 | [-18.92, 19.69] | 1 | 2731 | 7.45 | [-17.14, 31.72] | 1 | 3341 |
| HCT | 0.01 | [ -0.05, 0.06] | 1 | 3319 | 0.01 | [-0.06, 0.08] | 1 | 3213 |
| MAIA | 0.08 | [0.02, 0.14] | 1 | 4277 | -0.01 | [-0.08, 0.08] | 1 | 3974 |
| STAI-S | 0.33 | [0.07, 0.57] | 1 | 2912 | -0.27 | [-0.56, 0.03] | 1 | 3472 |
| CD:HCT | -1.09 | [-2.20, 0.05] | 1 | 3616 | -0.92 | [-2.31, 0.47] | 1 | 3895 |
| CD:MAIA | -0.67 | [-2.08, 0.75] | 1 | 2239 | 0.57 | [-1.16, 2.38] | 1 | 2769 |
| HCT:MAIA | 0.00 | [-0.00, 0.00] | 1 | 4603 | -0.00 | [-0.00, 0.00] | 1 | 4759 |
| CD:HCT:MAIA | 0.08 | [-0.00, 0.15] | 1 | 2562 | 0.01 | [-0.09, 0.11] | 1 | 3006 |

HCT, Heartbeat Counting Task score; MAIA, Multidimensional Assessment of Interoceptive Awareness; STAI-S, State-Trait Anxiety Inventory (State); HP, hemodynamic profile; CD, compensation deficit; 95% CI, 95% credible intervals; ESS, effective sample size

**Supplementary Table 2**. The interactive effects of compensation deficit and interoception on the evaluation of excitement and nervousness during the private speech task.

|  | Excitement | | | | Nervousness | | | |
| --- | --- | --- | --- | --- | --- | --- | --- | --- |
| Parameters | Estimate | 95%CI | R | ESS | Estimate | 95%CI | R | ESS |
| Intercept | 14.46 | [13.08, 15.84] | 1 | 5061 | 12.58 | [10.95, 14.31] | 1 | 4328 |
| CD | -13.00 | [-37.78, 12.49] | 1 | 3576 | 11.96 | [-16.91, 41.73] | 1 | 3930 |
| HCT | 0.02 | [ -0.04, 0.09] | 1 | 3667 | 0.04 | [-0.04, 0.12] | 1 | 3948 |
| MAIA | -0.00 | [-0.08, 0.07] | 1 | 3087 | 0.03 | [-0.06, 0.12] | 1 | 3889 |
| STAI-S | 0.17 | [-0.11, 0.44] | 1 | 4299 | 0.00 | [-0.30, 0.31] | 1 | 3932 |
| CD:HCT | 0.00 | [-1.01, 1.03] | 1 | 4088 | -0.18 | [-1.38, 1.04] | 1 | 4175 |
| CD:MAIA | 0.42 | [-0.80, 1.67] | 1 | 4105 | 0.27 | [-1.20, 1.68] | 1 | 3749 |
| HCT:MAIA | -0.00 | [-0.00, 0.00] | 1 | 4523 | 0.00 | [-0.00, 0.00] | 1 | 4149 |
| CD:HCT:MAIA | -0.01 | [-0.05, 0.03] | 1 | 3717 | 0.02 | [-0.02, 0.07] | 1 | 3785 |

HCT, Heartbeat Counting Task score; MAIA, Multidimensional Assessment of Interoceptive Awareness; STAI-S, State-Trait Anxiety Inventory (State); HP, hemodynamic profile; CD, compensation deficit; 95% CI, 95% credible intervals; ESS, effective sample size
